# Influence of Plant Additives on Antimicrobial Properties of Glass-Fabric-Reinforced Epoxy Composites Used in Railway Transport

**DOI:** 10.3390/ma17184666

**Published:** 2024-09-23

**Authors:** Aleksandra Węgier, Filip Kaźmierczyk, Magdalena Efenberger-Szmechtyk, Angelina Rosiak, Joanna Kałużna-Czaplińska, Anna Masek

**Affiliations:** 1 Institute of Polymer and Dye Technology, Faculty of Chemistry, Lodz University of Technology, Stefanowskiego 16, 90-537 Lodz, Poland; aleksandra.wegier@dokt.p.lodz.pl; 2 TAPS Maciej Kowalski, Borowa 4, 94-247 Lodz, Poland; 3 Department of Strength of Materials, Faculty of Mechanical Engineering, Lodz University of Technology, Stefanowskiego 1/15, 90-537 Lodz, Poland; filip.kazmierczyk@p.lodz.pl; 4 Institute of Fermentation Technology and Microbiology, Faculty of Biotechnology and Food Sciences, Lodz University of Technology, Wólczańska 171/173, 90-530 Lodz, Poland; magdalena.efenberger-szmechtyk@p.lodz.pl; 5 Institute of General and Ecological Chemistry, Faculty of Chemistry, Lodz University of Technology, Żeromskiego 116, 90-924 Lodz, Poland; angelina.rosiak@p.lodz.pl (A.R.); joanna.kaluzna-czaplinska@p.lodz.pl (J.K.-C.)

**Keywords:** epoxy resin composites, natural additives, phytochemicals, antimicrobial, railway seats

## Abstract

The aim of this research was to explore the innovative use of natural additives, containing phytochemicals with proven antimicrobial effects, in the production of epoxy–glass composites. This study was based on information regarding the antimicrobial effects of phytochemicals present in *Cistus incanus*, *Zingiber officinale*, and *Armoracia rusticana*. The additives were subjected to a gas chromatography (GC) analysis to determine their composition, and, subsequently, they were used to prepare resin mixtures and to produce epoxy–glass composites. Samples of the modified materials were tested against *E. coli*, *S. aureus*, and *C. albicans*. In addition, flammability and durability tests were also performed. It was found that the strongest biocidal properties were demonstrated by the material with the addition of cistus, which caused a reduction of microorganisms by 2.13 log units (*S. aureus*), 1.51 log units (*E. coli*), and 0.81 log units (*C. albicans*). The same material also achieved the most favorable results of strength tests, with the values of flexural strength and tensile strength reaching 390 MPa and 280 MPa, respectively. Public transport is a place particularly exposed to various types of pathogens. Currently, there are no solutions on the railway market that involve the use of composites modified in this respect.

## 1. Introduction

**Figure 1:**
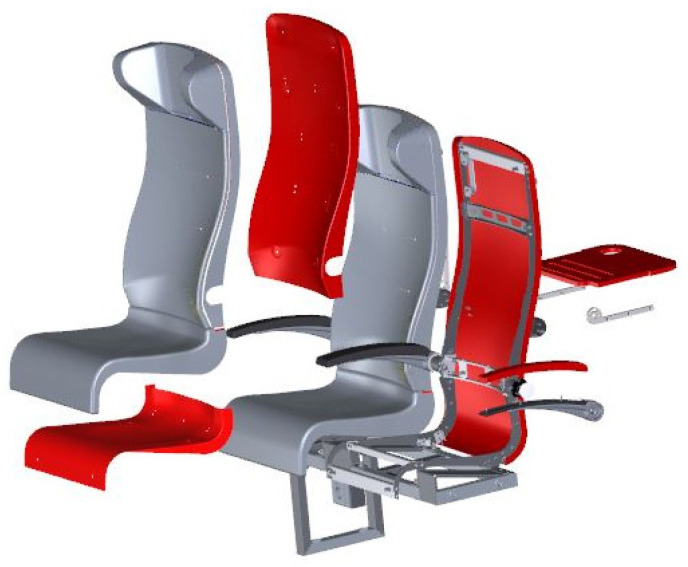
Visualization of a railway seat with polymer composite elements marked in red.

Railway seats are elements that have direct contact with passengers and, therefore, must provide them with not only comfort but, above all, safety. As a result, they are subjected to a number of requirements and standards that must be met. Currently, a lot of attention is paid to materials with antimicrobial properties. It is of great importance to use ecological, degradable materials and to follow the principles of sustainable development. One of the most important requirements in the railway industry is fire resistance. Each material, or combination of materials, used as an element of the railway seat must meet the requirements of the PN EN 45545-2 standard [1]. The use of polymeric materials as components of the seat is beneficial, especially due to their low weight, but significant problems regarding their flammability and residual waste after exploitation remain to be solved. Examples of seat elements for which polymeric materials may be used are presented in Figure 1, highlighted with the red color.

Epoxy resins are polymers with a very wide range of applications, which is due to their excellent properties, such as chemical resistance, mechanical strength, and good adhesion [2,3,4,5]. Combined with reinforcement fabrics, they create durable composite structures. By modifying the resin with antibacterial or flame-retardant additives, it is possible to obtain materials that meet the rigorous standards set for the railway industry. Nowadays, more and more attention is paid to the eco-friendliness of the materials used. As a result, raw materials of natural or recycled origins are becoming increasingly more popular. With the safety and comfort of passengers in mind, extra attention is also paid to the antimicrobial properties of the materials. Public transport is a place where passengers are particularly exposed to various types of microorganisms [6]. Antibacterial properties in epoxy resin can be obtained in several ways. Metallic nanoparticles or metal oxides (Ag, Cu, ZnO, TiO_2_) are often used, which, in low concentrations, exhibit antimicrobial activity due to their shape, size, charge, and high reactivity [7,8,9]. NPs affect pathogens in various ways, generally through adsorption and penetration of the cell wall [10]. They can cause damage to bacterial cells through the production of reactive oxygen species and free radicals, leading to oxidative stress [11]. Other possible mechanisms are the interaction with proteins and DNA, contributing to changes in their structure and function [12], or electrostatic binding to the bacterial cell wall, thus impairing the cell membrane [11]. Organic compounds are also successfully used to produce antibacterial composites, such as the hydrophilic biopolymer chitosan, along with quaternary ammonium compounds (QACs), which affect the bacterium by penetrating its cell membrane. An example of a QAC is cetylpyridinium, where the positively charged pyridine group attracts the negatively charged bacterial cell membrane. It is also worth mentioning carbon-based additives, such as carbon nanotubes, which easily penetrate the bacterial cell membrane, creating a network on the cell surface in solution and then destroying the bacterial envelope by leaking its contents. Antimicrobial effects can also be achieved by using clays, such as smectites (e.g., montmorillonite, vermiculite), which have the ability to adsorb and retain harmful, toxic substances. Additionally, clays can be modified, e.g., by adding metal cations, thus obtaining excellent antibacterial properties [7,8]. Taking into account ecological and economic approaches, in accordance with the principles of a sustainable economy, the use of plant materials containing phytochemicals, such as polyphenols, is an interesting approach. Polyphenols are famous for their antimicrobial and antioxidant properties. These substances belong to the group of secondary metabolites, i.e., those that do not directly participate in the growth, development, and reproduction of the plant. Their task is to protect the plant against harmful pathogens, parasites, toxins, and UV radiation and also to give the plant its smell, color, and taste. The group of secondary metabolites with antimicrobial properties includes phytochemicals, such as alkaloids, coumarins, flavonoids and isoflavonoids, essential oils and terpenoids, phenolic compounds, polyacetylenes, lignans, and xanthones [13]. Substances of plant origin are non-toxic, environmentally friendly, biodegradable, reasonably priced, and easily available. They may come from various parts of plants, e.g., the root, stem, leaves, or seeds. Recently, secondary metabolites have become more prominent, especially in terms of their usage in the food, cosmetics, and pharmaceutical industries [14]. For ecological reasons, plant substances are also tested in the polymer industry. Many scientific research reports mention plant extracts, among others, as anti-corrosion and antibacterial additives, stabilizers, and hardeners [15,16,17,18,19]. This is considered a safe alternative to synthetic antibacterial additives, such as nanosilver, or other metal compounds that may be harmful to human health. The popularity of metallic NPs has led to considerations regarding the risks associated with their use, such as their potential toxicity and environmental impact [20,21,22,23,24].

Substances that protect plants against harmful microorganisms include polyphenols, which consist of at least one hydroxyl group connected to an aromatic benzene ring. It is the hydroxyl group that is responsible for interactions with microbial cells [15]. 

So far, no composites with plant additives have been used in railway seats, which is an opportunity for the company to introduce an innovative product to the market. Epoxy–glass composites are an interesting material that is a suitable replacement in some elements of a railway seat’s structure, thus guaranteeing sufficient mechanical strength and low weight. Due to the possibility of modifying the polymer matrix, it is also possible to give the seat components special features, e.g., antibacterial properties, thus increasing the safety of passengers in mass transport. It is worth looking for a safe alternative to nanosilver and nanocopper, which are often used as antibacterial agents. In this study, substances of plant origin—cistus, horseradish, and ginger—were added to epoxy resin and used to produce glass-fibre-reinforced epoxy composites intended as parts of railway seats with increased antimicrobial properties.

Horseradish (*Armoracia rusticana*) is a plant which originates from Eastern Europe and southwestern Russia. It has been used since ancient times as a medical plant and as a spice. The medicinal part of horseradish is its root [25]. *A. rusticana*, which also occurs in Poland, is a perennial plant with significant antibacterial and antifungal properties [26,27,28]. These properties led to the idea of using it in the production of biostatic composites.

Glucosinolates are responsible for the biological activity of *A. rusticana*. This is a class of secondary plant metabolites that occur in dicotyledonous plants in the order Capericaceae [28]. Glucosinolates are S-glycosides containing sulfur and a glucose molecule in their structure [25]. Among the glucosinolates present in horseradish root, the majority are sinigrin [29], glucobrassicin, neoglucobrassicin, and gluconasturin [30]. After the disruption of plant tissue during the processing of horseradish root (e.g., grinding, cutting), glucosinolates are released, which are believed to be located in the cell vacuole. They are hydrolyzed by the enzyme myrosinase (thioglucoside glucohydrolase), which is located in the cytoplasm. Myrosinase hydrolytically separates the glucose molecule, contributing to the formation of an unstable intermediate, aglycone, which spontaneously transforms into isothiocyanate, nitrile, or thiocyanate. The type of product obtained depends on the reaction conditions (substrate, pH, temperature, presence of ferrous ions) [28]. Isothiocyanates have been the subject of various studies in the context of antibacterial [31] and antifungal activity. Park et al. examined the activity of isothiocyanates obtained from horseradish root against several types of pathogens, including *S. aureus* and the fungus *C. albicans.* Horseradish root extracts showed activity against all microorganisms, including, in particular, *C. albicans*, with MIC values of 0.52 ± 0.18 mg/mL and an MBC of 1.25 ± 0.00 mg/mL [27]. Mucete et al. inspected the activity of horseradish extracts against *E. coli*, *C. albicans*, and *A. niger*. The study showed notable activity against *E. coli* and *C. albicans*. In the case of *E. coli*, the response of microorganisms to higher concentrations of isothiocyanate (from 3.183 to 4.653 mmol) ranged from 0.5 cm to 1 cm of microorganism-free zones. For *C. albicans*, zones without microorganisms reached diameters of 0.4 cm to 1 cm at concentrations of isothiocyanate from 3.781 mmol to 3.368 mmol [28].

Cistus (*Cistus incanus*) is a plant found in the Mediterranean region that is also called “rock rose” [14]. Its medicinal use was initially associated with the production of the labdanum resin, which contains various types of phytochemicals [32]. 

Cistus is rich in polyphenols, especially catechins, which enable it to survive in diverse, often extreme environmental conditions. It contains various classes of secondary metabolites, including flavonoids, coumarins, terpene derivatives, and hydrocarbons [33].

Ginger (*Zingiber officinale*) is a plant from China that is nowadays also found in India, southwestern Asia, and western Africa [34]. It is associated primarily with a spice commonly used in cuisines around the world. Ginger contains biologically active substances, such as phenols, including gingerols, shogaols, and zingerones, and terpenes, including β-Bisabolene, α-Curcumene, zingiberene, α-Farnesene, and β-Sesquiphellandrene [35].

As part of this research, selected plant additives were subjected to GC analysis to determine the content of phytochemicals. Then, samples of composites with these additives were prepared, and their impact on the antimicrobial properties, flammability, and strength of the composites was determined.

## 2. Materials and Methods

### 2.1. Materials 

Plant substances were used in the form of powdered horseradish (the countries of origin were Poland and China), ginger (the countries of origin were Nigeria and Peru), and cistus (the country of origin was Turkey). All additives were produced by P.W. Rekord Józef Szwajkowski (Borków Stary 11, 62–817 Żelazków, Poland). The composites consisted of glass fabric reinforcement (2/2 twill weave, 350 g/m^2^, Rymatex, Rymanów, Poland) and a polymer matrix of flame-retardant epoxy resin (NEMresin 1011, New Era Materials, Modlniczka, Poland). The symbols and compositions of the individual composites are listed in Table 1.

### 2.2. Modification of the Resin Mixture 

The plant additives were mixed with the resin composition using a laboratory mixer, type RZR1 (Heidolph Instruments Gmbh & Co. KG, Schwabach, Germany). The mixing parameters were 2000 rpm for 10 min.

### 2.3. Preparation of the Composites 

The resin mixture was filtered through glass fabric at a pressure of 2.2 bar and a temperature of 88 °C to form a prepreg. Then, the prepregs were cross-linked under a vacuum membrane press (Wilking) at a temperature of 130 °C for 30 min.

### 2.4. Methods

#### 2.4.1. Gas Chromatography

Solid samples were extracted with ethanol. Extraction was carried out in PP tubes (5 mL) for 15 min with the use of the rotator (Grant Bio PTR-30, Grant Instruments (Cambridge) Ltd., Royston, UK). After centrifugation (Hettich EBA 21, Andreas Hettich GmbH & Co. KG, Tuttlingen, Germany), the obtained ethanol fractions were analyzed using a gas chromatograph–mass spectrometer with a time-of-flight analyzer (GC–TOFMS). Analyses were carried out using an Agilent 7890B Gas Chromatograph (Agilent Technologies, Santa Clara, CA, USA) equipped with an Rxi-5MS capillary column (30 m × 250 μm × 0.25 μm) coupled to a LECO Pegasus BT time-of-flight detector (LECO Corporation, St. Joseph, MI, USA). The inlet temperature was kept at 250 °C. The column oven was programmed for the temperature at a starting point of 100 °C for 5 min. Then, the temperature was increased by 15 °C per minute until it reached 300 °C. The final temperature was kept for 3 min. The carrier gas (helium) flow was at a constant flow rate of 1.0 mL/min. The mass range was set between *m*/*z* 50 and 650.

The compounds were identified using the NIST14 spectra library. This study included compounds that had a content over 0.1%

#### 2.4.2. Assessment of Antimicrobial Activity

In order to determine the antimicrobial activity of the prepared composite samples, we applied a method in accordance with the ASTM E2180 [36] (standard test method for determining the activity of incorporated antimicrobial agent(s) in polymeric or hydrophobic materials). The tests were performed on square samples measuring 2 × 2 cm. The specimens were treated with ethanol to disinfect them and with distilled water to remove alcohol residues. Afterwards, they were placed in a sterile dish. Microorganisms from the American-Type Culture *Collection* (*ATCC*) were used for the study, including the Gram-negative bacteria *Escherichia coli* ATCC 10536, the Gram-positive *Staphylococcus aureus* ATCC 6538, and fungi in the form of the yeast *Candida albicans* ATCC 10231. Merck media were used for the tests, including TSA for bacteria and MEA for fungi. The organisms were stored at 6 °C and activated before the start of composite testing. A suspension of microorganisms was prepared in physiological saline with the addition of 0.3% agar. Each composite was covered with a suspension of microorganisms in the amount of 0.2 mL. Microbiological analysis was performed immediately after applying the suspension (*t* = 0 h) and after 24 h of incubation (*t* = 24 h). The microbial suspension was rinsed with 10 mL of neutralizer. Then, the number of microorganisms was determined using the culture method in TSA (bacteria) and MEA (yeast) media. The dieback rate of microorganisms was calculated using the following equation:

D = (log number of microorganisms*_t_*_= 0_ − log number of microorganisms*_t_*_= 24 h_) (1)

The results were expressed as the number of colony-forming units per 1 cm^2^ of material.

#### 2.4.3. Flammability Tests

Flammability tests were carried out in accordance with the R6 requirement of PN EN 45545-2 [1]. The maximum average heat release intensity (MARHE) was determined in accordance with ISO 5660-1 [37], and smoke production was determined in accordance with PN-EN ISO 5659-2 [38]. For MARHE tests, three plates with dimensions of 100 × 100 × 2.5 mm were cut. Samples measuring 75 × 75 × 2.5 mm were prepared for smoke-production tests. The samples were seasoned in a climatic chamber for 48 h at a temperature of 23 ± 2 °C and a humidity of 50 ± 5%.

#### 2.4.4. Hardness Measurements

Hardness measurements were performed using a PosiTector gauge (Defelsko, Ogdensburg, NY, USA) with a Barcol scale probe. Measurements were taken using samples with dimensions of 500 × 500 × 2.5 mm, with 5 measurement repetitions at intervals of at least 2 cm.

#### 2.4.5. Material Strength Tests

The static tensile test was carried out on the 50 kN Universal Testing Machine (Shimadzu, Kyoto, Japan), in accordance with the PN-EN ISO 527-4 standard [39]. The Zwick/Roell Z005 apparatus (Ulm, Germany) was used for three-point bending tests, and the tests were carried out in accordance with the PN-EN ISO 178 [40] standard. Young’s modulus, the tensile strength, and the bending strength were determined.

## 3. Results and Discussion

### 3.1. GC Analysis of Plant Extracts

The GC analysis of the powdered horseradish root extract showed the presence of substances that may affect the antimicrobial properties (Table 2).

**Figure 2:**
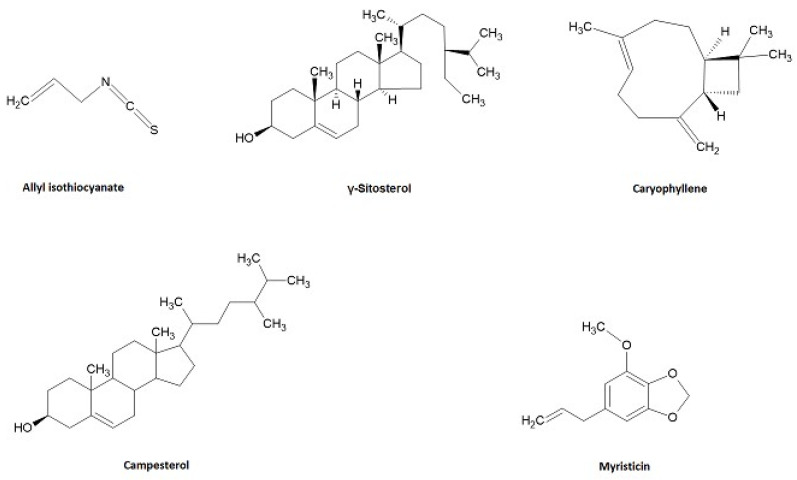
Chemical structures of the main active substances in *A. rusticana* [43].

The most significant chemical compounds in terms of antibacterial use are isothiocyanates, including, in particular, allyl isothiocyanate, the activity of which is described in several studies [41,42]. The tested extract also contained the presence of myristicin, which belongs to the phenylpropanoid group, and single terpenoids, including Caryophyllene, Campesterol, and γ-Sitosterol. The chemical structures of the main active substances found in horseradish are shown in Figure 2.

**Figure 3:**
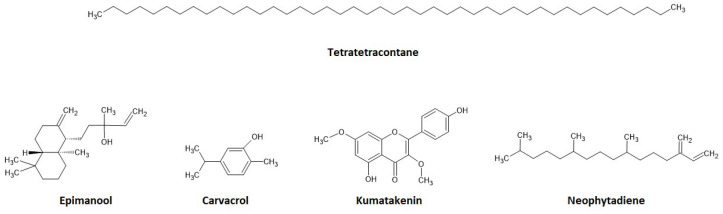
Chemical structures of the main active substances in *C. incanus* [43].

After a GC analysis of the cistus extract, active substances were identified (Table 3), including a significant amount of tetratetracontane, a long-chain, unbranched alkane with the chemical formula C_44_H_90_, which has confirmed antimicrobial properties [44,45,46,47]. In addition, thirty different substances from the terpenoid group were identified, including Epimanool, Carvacrol, and Neophytadiene (Figure 3). Their activity against pathogens has been documented in numerous studies [32,48,49]. Eight different flavonoids, four alkaloids, and five phenols were also recognized.

**Figure 4:**
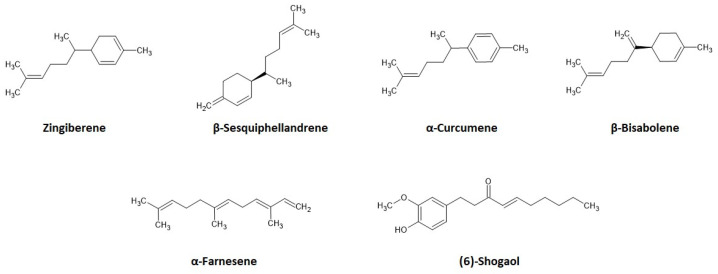
Chemical structures of the main active substances in *Z. officinale* [43].

A total of twenty-eight terpenoids were detected in the tested ginger extract (Table 4), including mainly sesquiterpenoids, as follows: Zingiberene (area 15.34%), β-Sesquiphellandrene (area 7.27%), α-Curcumene (area 5.33%), β-Bisabolene (area 3.89%), and α-Farnesene (area 2.28%). The chemical structures of the mentioned substances are shown in Figure 4. The analysis allowed for the identification of sixteen phenolic compounds, mostly shogaols, which are formed as a result of drying or heating gingerols and are responsible, in particular, for the taste of ginger [34,35]. There are studies in the literature on their antibacterial activity, including ingredients, such as zinigiberene, α-Farnesene, 6-gingerol, and α-Curcumene. They are supposed to influence the permeability and release of the intracellular components of bacteria by attacking membranes and cell walls [50,51].

### 3.2. Assessment of Antimicrobial Properties of Composites with Plant Additives

The tested plant substances were used as an addition to the epoxy matrix of composites reinforced with glass fabric. The prepared samples were subjected to microbiological tests. The test results are presented in Table 5.

Among the tested composites, the strongest biocidal properties were demonstrated by the material with the addition of cistus, which had an effect on both Gram-negative and Gram-positive bacteria, and, to a slightly lesser extent, on fungi. It caused a reduction in microorganisms by 2.13 (*S. aureus*), 1.51 (*E. coli*), and 0.81 (*C. albicans*) log units. The composite sample with the addition of ginger only had a biocidal effect against the Gram-negative bacteria *E. coli*. Only slight antimicrobial activity against *E. coli* was recorded for composites with the addition of horseradish. This may be due to the easy degradation of isothiocyanates, especially under the influence of temperature [52]. Allyl isothiocyanate may also react with amines, i.e., hardeners used in epoxy resin [53].

### 3.3. The Influence of Plant Additives on the Flammability Properties of Composites

In the railway industry, some of the most stringent requirements to be met are those relating to fire protection. Each material used in a train is subject to a flammability assessment in accordance with guidelines that vary depending on the function of the material, its mass, or its location. The materials used as elements of the backrest support, located at the back of the seat, are subject to requirement R6 of the PN-EN 45545-2 standard (see Table 6), which assumes, among other things, the need to perform in accordance with the Maximum Average Rate of Heat Emission (MARHE, ISO 5660-1) and smoke emission (PN-EN ISO 5659-2). The assessed parameters in the case of backrest carriers are the optical density of smoke after the first 4 min of the test (Ds_4) and the area under the optical density graph after the first 4 min of the test (VOF_4).

**Figure 5:**
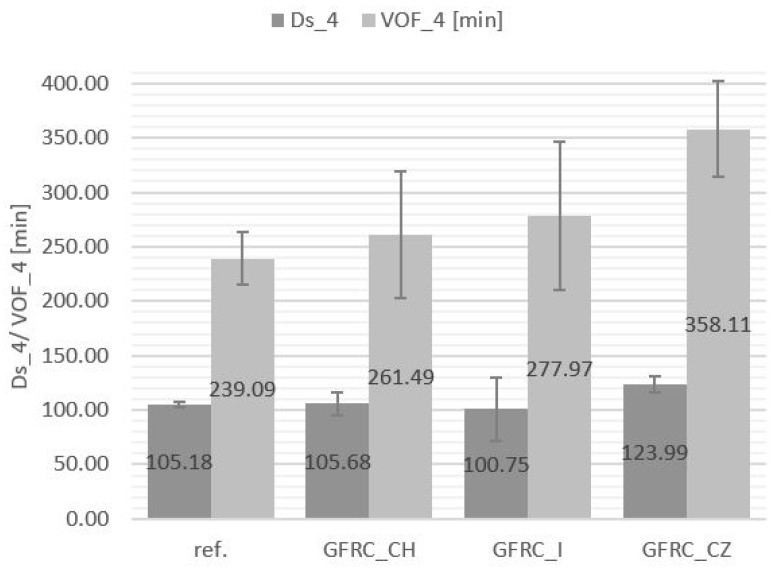
Results of smoke emission tests of composites: reference sample (Ref.), with the addition of horseradish (GFRC_CH), ginger (GFRC_I), and cistus (GFRC_CZ).

**Figure 6:**
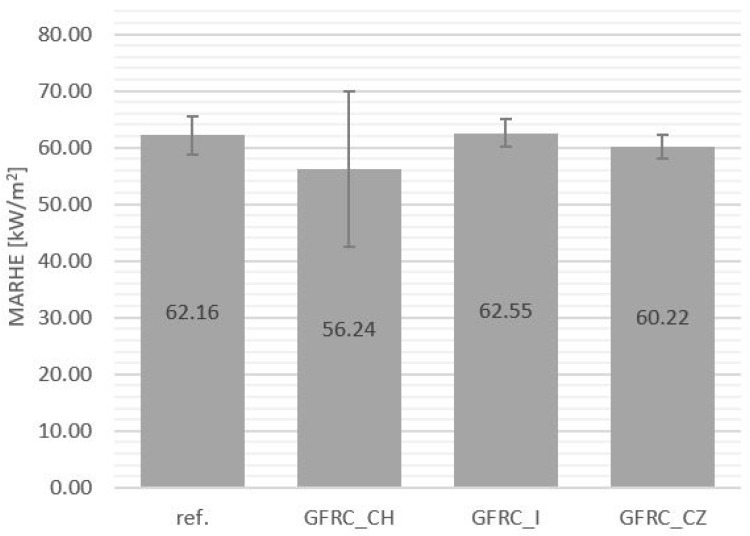
Test results—Maximum Average Rate of Heat Emission (MARHE): reference sample (Ref.), with the addition of horseradish (GFRC_CH), ginger (GFRC_I), and cistus (GFRC_CZ).

Composites with plant additives did not show significant differences in the MARHE value compared to the unfilled reference sample (62.16 kW/m^2^), while a slight decrease in this value was recorded for the sample with the addition of horseradish (56.24 kW/m^2^). Smoke-production parameters were also more favorable in the case of the GFRC_CH when compared to other composites filled with plant additives. The values of smoke emission and MARHE of the tested materials are presented in Figure 5 and Figure 6, respectively. Each material meets the R6 requirements of the PN-EN 45545-2 standard [1] at the HL2 level and is suitable for the production of railway seat components.

### 3.4. The Influence of Plant Additives on the Mechanical Properties of Composites

**Figure 7:**
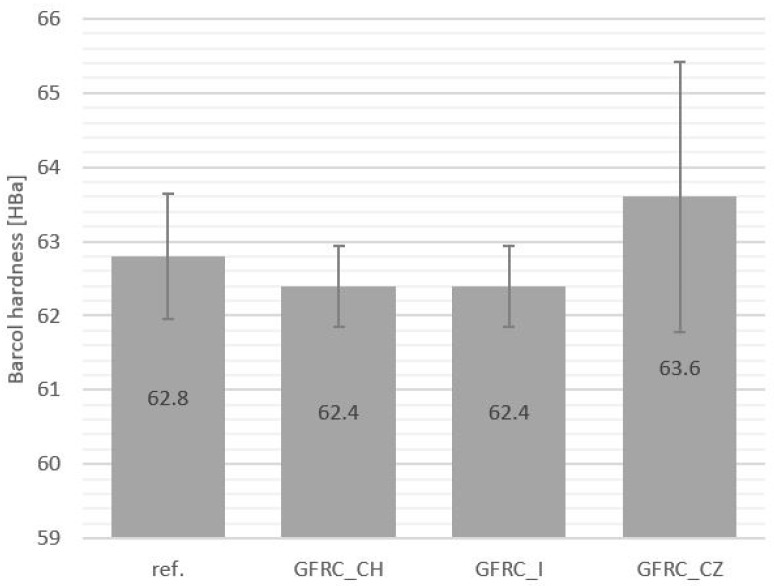
Barcol hardness test results: reference sample (Ref.), with the addition of horseradish (GFRC_CH), ginger (GFRC_I), and cistus (GFRC_CZ).

**Figure 8:**
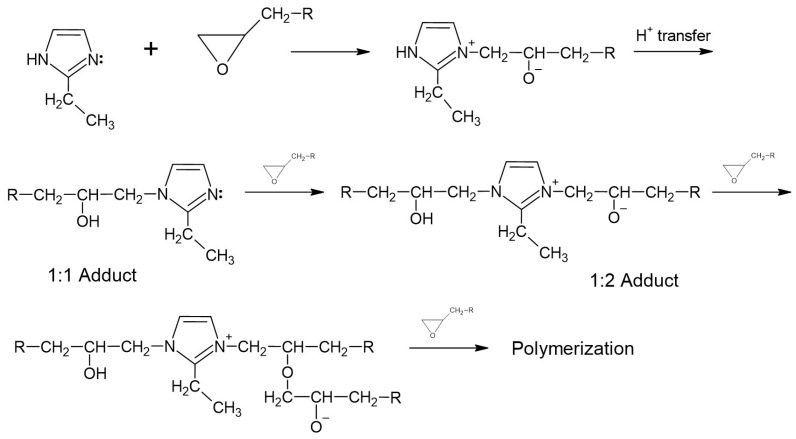
Reaction scheme for hardening epoxy resins using 2-ethylimidazole [54].

The tests did not show any significant changes in the hardness of samples filled with additives compared to the reference material. A slightly higher average Barcol hardness was recorded for the material with the addition of cistus (Figure 7). The lack of changes in Barcol hardness compared to the well-cross-linked reference sample indicates an appropriate degree of cross-linking of the composites. The hardener intended to cure the epoxy resin used during the preparation of composites is 2-ethylimidazole. Cross-linking with imidazoles takes place gradually (Figure 8). First, the epoxy group reacts with the nitrogen in the pyridine ring present in the hardener structure. Then, the hydrogen transfer occurs, which causes the second nitrogen atom to change from pyrrole to pyridine, which is capable of reacting with another epoxy group, thus creating an adduct with both a positive and a negative charge. Polymerization occurs using a negative charge [54].

**Figure 9:**
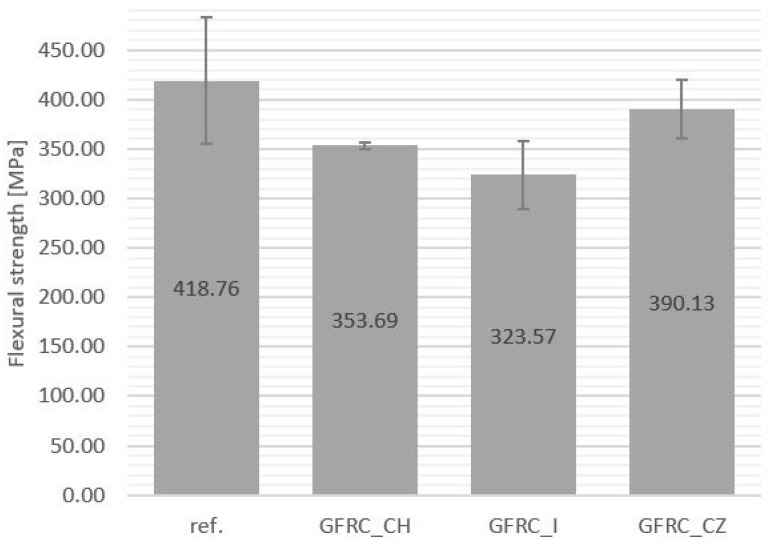
Three-point bending strength values: reference sample (Ref.), with the addition of horseradish (GFRC_CH), ginger (GFRC_I), and cistus (GFRC_CZ).

Plant additives present in the composite matrix resulted in a reduction in bending stress in the case of samples containing horseradish and ginger (Figure 9). For the sample with cistus, a slight increase in this value was noted. This may be caused by a lower number of defects, less delamination, and better resin infiltration of the GFRC_CZ sample compared to other materials with plant additives. The reason for the deterioration of mechanical properties may be a problem with the wettability of fillers and their distribution in the polymer matrix. Numerous scientific publications indicate the deterioration of the strength results of composites due to the poor dispersion of powder fillers [55].

**Figure 10:**
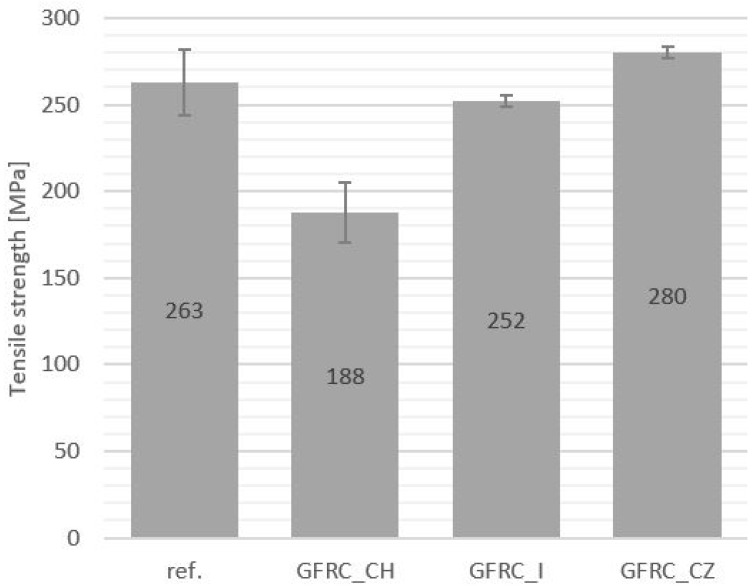
Tensile strength values: reference sample (Ref.), with the addition of horseradish (GFRC_CH), ginger (GFRC_I), and cistus (GFRC_CZ).

**Figure 11:**
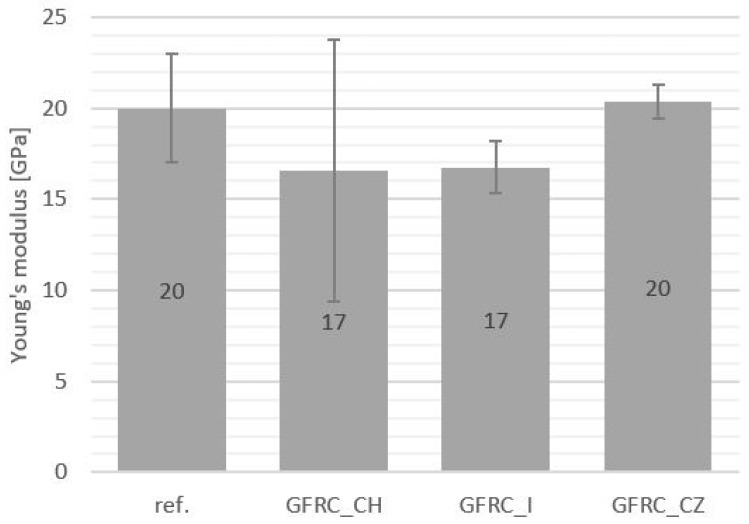
Young’s modulus values: reference sample (Ref.), with the addition of horseradish (GFRC_CH), ginger (GFRC_I), and cistus (GFRC_CZ).

The results of the static tensile test also indicate a slight weakening of the composites with the addition of ginger and horseradish. The material with the addition of cistus achieved faintly higher tensile strength values than the unfilled sample (see Figure 10). However, this is a negligible value and only proves the lack of negative impact of this additive on the structure of the composite. In the case of Young’s modulus (Figure 11), the values for the cistus composite sample and the reference material did not differ. The remaining additives caused a decrease in the Young’s modulus by 3GPa compared to the reference representative.

The addition of powder fillers, including both plant additives and flame retardants, changes the viscosity of the resin, making it difficult to wet the glass reinforcement and get rid of the gas, thus causing delamination, empty spaces, and air bubbles in the structure of the material. This may significantly affect the strength parameters depending on the type of added substance, the grain size, or the hygroscopicity. According to literature reports, the presence of -OH groups on the surface of fillers can also cause a catalytic effect, thus increasing the degree of cross-linking of the polymer [55]. This may be the reason for higher values of strength parameters for samples with the addition of cistus, which contains a significant amount of substances containing -OH groups.

## 4. Conclusions

The results of the conducted research showed that the use of horseradish powder as an addition to powdered epoxy resin did not have a positive effect on the antimicrobial properties and contributed to the deterioration of the mechanical properties of the composite. Despite the detection of allyl isothiocyanate in the tested extract, which had a proven effect on microorganisms, the composite made with this additive did not exhibit any effect on bacteria and fungi, which could be the result of the low durability of this compound and a lack of resistance to the resin-processing temperature. The material with the addition of ginger expressed a minor effect on the number of *E. coli* bacteria but no effect on other microorganisms. A beneficial outcome of increasing the antimicrobial properties of the epoxy–glass composite occurred in the case of the addition of cistus. The GC analysis of this compound indicated the highest content of various types of terpenoids, alkaloids, phenols, and hydrocarbons among all of the tested additives. Substances of natural origin often contribute to greater susceptibility to ignition, but, in the case of flame-retardant composites, the negative impact of plants on flammability remained insignificant. Each of the materials prepared in this study is suitable for usage in the railway industry in terms of flammability requirements. Taking into account the small impact on the mechanical strength and the favorable results of other tests, the addition of cistus is an interesting possibility for modifying composites, and it is suitable for conducting substantial numbers of more detailed tests.

## Figures and Tables

**Table 1:** Symbols and compositions of the prepared composite samples.

Symbol	Composition
Ref.	Glass-fibre-reinforced epoxy composite [GFRC]
GFRC_CH	GFRC + *A. rusticana* powder
GFRC_I	GFRC + *Zingiber officinale* powder
GFRC_CZ	GFRC + *Cistus incanus* powder

**Table 2:** Main active ingredients in *A. rusticana* obtained through GC analysis.

Isothiocyanate	R.T. (min)	Area [%]
Allyl Isothiocyanate	3.77	1.78
Benzene, (2-isothiocyanatoethyl)-	9.48	1.56
4-Methoxyphenyl isothiocyanate	8.44	0.44
**Terpenoid**		
γ-Sitosterol	23.00	0.58
Caryophyllene	9.09	0.24
Campesterol	21.84	0.22
**Phenylpropanoid**		
Myristicin	9.85	0.62

**Table 3:** Main active ingredients in *C. incanus* obtained through GC analysis.

Terpenoid	R.T. (min)	Area %	Other Name
1-Naphthalenepropanol, α-ethenyldecahydro-5-(hydroxymethyl)-α,5,8a-trimethyl-2-methylene-, [1S-[1α(S*),4aβ,5α,8aα]]-	15.75	2.98	Epimanool
1-Phenanthrenemethanol, 1,2,3,4,4a,9,10,10a-octahydro-1,4a-dimethyl-7-(1-methylethyl)-, [1S-(1α,4aα,10aβ)]-	15.62	1.31	Abietyl alcohol
Phenol, 2-methyl-5-(1-methylethyl)-	7.88	1.18	Carvacrol
1,3-Butadiene, 2-(4,8,12-trimethyltridecyl)-	12.12	0.90	Neophytadiene
γ-Sitosterol	23.02	0.88	Clionasterol
dl-α-Tocopherol	20.50	0.80	6-Chromanol
3,7,11,15-Tetramethyl-2-hexadecen-1-ol	13.94	0.65	Phytol
7-Isopropyl-1,1,4a-trimethyl-1,2,3,4,4a,9,10,10a-octahydrophenanthrene	13.83	0.60	
1H-Naphtho [2,1-b]pyran, 3-ethenyldodecahydro-3,4a,7,7,10a-pentamethyl-, [3R-(3α,4aβ,6aα,10aβ,10bα)]-	13.46	0.45	Manoyl oxide
1-Naphthalenepropanol, α-ethenyldecahydro-2-hydroxy-α,2,5,5,8a-pentamethyl-, [1R-[1α(R*),2β,4aβ,8aα]]-	16.39	0.41	Sclareol
Urs-12-en-3-ol, (3β)-	24.47	0.41	α-Amyrin
Olean-12-en-3-ol, (3β)-	23.74	0.41	β-Amyrin
2-Phenanthrenol, 4b,5,6,7,8,8a,9,10-octahydro-4b,8,8-trimethyl-1-(1-methylethyl)-, (4bS-trans)-	15.57	0.32	Totarol
1-Phenanthrenemethanol, 1,2,3,4,4a,9,10,10a-octahydro-1,4a-dimethyl-7-(1-methylethyl)-, [1R-(1α,4aβ,10aα)]-	15.34	0.29	Abietyl alcohol
2H-1-Benzopyran-6-ol, 3,4-dihydro-2,7,8-trimethyl-2-(4,8,12-trimethyltridecyl)-	19.72	0.27	γ-Tocopherol
(-)-Bicyclo [2.2.1]heptan-2-one, 1,3,3-trimethyl-	5.86	0.26	L-Fenchone
1-Phenanthrenecarboxaldehyde, 1,2,3,4,4a,9,10,10a-octahydro-1,4a-dimethyl-7-(1-methylethyl)-, [1S-(1α,4aα,10aβ)]-	15.08	0.26	Dehydroabietal
Bicyclo [2.2.1]heptan-2-one, 1,7,7-trimethyl-, (1S)-	6.46	0.24	Camphor
Naphthalene, 1,2,3,4-tetrahydro-1,6-dimethyl-4-(1-methylethyl)-, (1S-cis)-	9.92	0.24	Calamenene
Androstan-3-ol, (3β,5α)-	15.69	0.15	Androstanol
Cyclopentane, 1,2-dimethyl-3-(1-methylethyl)-	16.07	0.15	
2-Pentadecanone, 6,10,14-trimethyl-	12.18	0.14	Hexahydrofarnesyl acetone
4,7-Methanoazulene, 1,2,3,4,5,6,7,8-octahydro-1,4,9,9-tetramethyl-, [1S-(1α,4α,7α)]-	16.32	0.13	β-Patchoulene
1H-Naphtho [2,1-b]pyran-8(4aH)-one, 3-ethenyldecahydro-3,4a,7,7,10a-pentamethyl-	14.99	0.12	Epimanoyl oxide
1-((1S,3aR,4R,7S,7aS)-4-Hydroxy-7-isopropyl-4-methyloctahydro-1H-inden-1-yl)ethanone	11.62	0.11	Oplopanone
Naphtho [2,1-b]furan-2(1H)-one, decahydro-3a,6,6,9a-tetramethyl-	14.02	0.11	Sclareolide
Cyclohexene, 1-methyl-4-(1-methylethenyl)-, ds.-	5.22	0.11	D-Limonene
Benzene, 1-methyl-2-(1-methylethyl)-	5.17	0.11	o-Cymene
Ergost-5-en-3-ol, (3β,24R)-	21.85	0.11	Campesterol
Azulene, 1,2,3,5,6,7,8,8a-octahydro-1,4-dimethyl-7-(1-methylethenyl)-, [1S-(1α,7α,8aβ)]-	15.80	0.10	α-Bulnesene
**Flavonoid**			
4H-1-Benzopyran-4-one, 5-hydroxy-2-(4-hydroxyphenyl)-3,7-dimethoxy-	19.94	1.05	Kumatakenin
Phenol, p-(2-methylallyl)-	16.93	0.61	
2-(3-Hydroxy-4-methoxyphenyl)-3,7-dimethoxy-4H-chromen-4-one	19.23	0.9	3′-Hydroxy-’,4′,7-trimethoxyflavone
4H-1-Benzopyran-4-one, 2,3-dihydro-5-hydroxy-2-(4-hydroxyphenyl)-7-methoxy-, (S)-	17.86	0.19	Sakuranetin
Quercetin-3,’,3′,4′-tetramethyl ether	20.79	0.13	Retusine
2-(4-Hydroxyphenyl)-3,6,7-trimethoxy-5-hydroxy-4H-1-benzopyran-4-one	20.53	0.11	Penduletin
Benzofuran, 2,3-dihydro-	7.14	0.59	Coumaran
4H-1-Benzopyran-4-one, 5-hydroxy-7-methoxy-2-(4-methoxyphenyl)-	19.08	0.58	Tectochrysin
**Alkaloid**			
Pyrazine, 2,3-dimethyl-5-(1-propenyl)-, (E)-	9.05	0.48	
Pyridine, 2-ethyl-6-methyl-	23.79	0.46	
Oxazolidine, 2,2-diethyl-3-methyl-	4.95	0.25	
Hydrazine, 1,1-diethyl-2-(1-methylpropyl)-	7.96	0.25	
**Phenol**			
(E)-4-(3-Hydroxyprop-1-en-1-yl)-2-methoxyphenol	11.55	0.21	(E)-Conipheryl alcohol
Benzeneacetic acid, 4-hydroxy-3-methoxy-	10.87	0.20	Homovanillic acid
1,2,3-Benzenetriol	8.77	0.91	Pyrogallol
2-Methoxy-4-vinylphenol	8.05	0.33	Varamol
Benzaldehyde	4.58	0.32	Artificial Almond Oil
**Hydrocarbone**			
Tetratetracontane	18.29	6.04	

**Table 4:** The main active ingredients in *Z. officinale* obtained through GC analysis.

Terpenoid	R.T. (min)	Area %	Other Name
1,3-Cyclohexadiene, 5-(1,5-dimethyl-4-hexenyl)-2-methyl-, [S-(R*,S*)]-	9.60	15.34	Zingiberene
Cyclohexene, 3-(1,5-dimethyl-4-hexenyl)-6-methylene-, [S-(R*,S*)]-	9.86	7.27	β-Sesquiphellandrene
Benzene, 1-(1,5-dimethyl-4-hexenyl)-4-methyl-	9.51	5.33	α-Curcumene
Cyclohexene, 1-methyl-4-(5-methyl-1-methylene-4-hexenyl)-	9.72	3.89	β-Bisabolene
1,3,6,10-Dodecatetraene, 3,7,11-trimethyl-	9.65	2.28	α-Farnesene
Naphthalene, 1,2,3,4,4a,5,6,8a-octahydro-7-methyl-4-methylene-1-(1-methylethyl)-, (1α,4aβ,8aα)-	9.75	1.72	α-Muurolene
2-Naphthalenemethanol, decahydro-α,α,4a-trimethyl-8-methylene-, [2R-(2α,4aα,8aβ)]-	10.99	0.60	β-Eudesmol
Bicyclo [2.2.1]heptan-2-ol, 1,7,7-trimethyl-, (1S-endo)-	6.66	0.57	L-Borneol
(1R,4R)-1-methyl-4-(6-Methylhept-5-en-2-yl)cyclohex-2-enol	10.58	0.52	Zingiberenol
1,6,10-Dodecatrien-3-ol, 3,7,11-trimethyl-	10.12	0.47	Nerolidol 2
Diepicedrene-1-oxide	12.03	0.42	
7,11-dimethyl-3-methylene-	9.22	0.41	cis-β-Farnesene
3-Cyclohexene-1-methanol, α,α4-trimethyl-	6.87	0.39	α-Terpineol
Longipinocarveol, trans-	22.68	0.34	
(I12Z)-(E)-3,7-Dimethylocta-2,6-dien-1-yl octadeca-9,12-dienoate	18.55	0.32	Geranyl linoleate
2H-1-Benzopyran-6-ol, 3,4-dihydro-2,7,8-trimethyl-2-(4,8,12-trimethyltridecyl)-	19.72	0.31	γ-Tocopherol
Humulenol-II	24.31	0.28	
Naphthalene, 1,2,3,4,4a,5,6,8a-octahydro-4a,8-dimethyl-2-(1-methylethylidene)-	9.93	0.28	Selina-3,7(11)-diene
5-(2,6-Dimethylbicyclo [3.1.1]hept-2-en-6-yl)-2-methyl-2-penten-1-ol-	12.45	0.26	Z-α-trans-Bergamotol
Bicyclo [3.1.0]hexan-2-ol, 5-(1,5-dimethyl-4-hexenyl)-2-methyl-	10.40	0.22	trans-Sesquisabinene hydrate
Naphthalene, 1,2,3,5,6,8a-hexahydro-4,7-dimethyl-1-(1-methylethyl)-, (1S-cis)-	9.89	0.20	δ-Cadinene
1,6,10,14-Hexadecatetraen-3-ol, 3,7,11,15-tetramethyl-, (E,E)-	14.26	0.19	Geranyl Linalool
ricyclo [4.4.0.02,7]dec-3-ene, 8-isopropyl-1,3-dimethyl-, (1R,2S,6S,7S,8S)-(-)-; (-)-α-Copaene	8.66	0.18	Copaene
Bicyclo [7.2.0]undec-4-ene, 4,11,11-trimethyl-8-methylene-, [1R-(1R*,4E,9S*)]-	9.28	0.16	Caryophyllene
Phenol, 5-(1,5-dimethyl-4-hexenyl)-Iethyl-, (R)-	11.58	0.16	Xanthorrhizol
Naphthalene, 1,2,3,4-tetrahydro-1,6-dimethyl-4-(1-methylethyl)-, (1S-cis)-	9.92	0.14	Calamenene
(1R,4aR,7R,8aR)-7-(2-Hydroxypropan-2-yl)-1,4a-dimethyldecahydronaphthalen-1-ol	12.19	0.13	Cryptomeridiol
Stigmast-5-en-3-ol, (3β,24S)-	23.01	0.13	γ-Sitosterol
Benzene, 1-(1,5-dimethyl-4-hexenyl)-4-methyl-	23.93	0.11	Curcumene
**Alkaloid**			
1H-Purine-2,6-dione, 3,7-dihydro-1,3,7-trimethyl-	12.38	1.69	Caffeine
6-Nonenamide, N-[(4-hydroxy-3-methoxyphenyl)methyl]-8-methyl-	16.56	0.50	Capsaicin
**Phenol**			
1-(4-Hydroxy-3-methoxyphenyl)dec-4-en-3-one	15.10	15.29	(6)-Shogaol
1-(4-Hydroxy-3-exadichenyl)tetradec-4-en-3-one	17.36	5.04	(10)-Shogaol
1-(4-Hydroxy-3-methoxyphenyl)dodec-4-en-3-one	16.24	2.78	(8)-Shogaol
Phenol, 2-methoxy-4-propyl-	14.03	1.74	DiIroeugenol
(E)-1-(4-Hydroxy-3-methoxyphenyl)dec-3-en-5-one	14.70	1.47	[6]-Isoshogaol
(E)-1-(4-Hydroxyexadicxyphenyl)tetradec-3-en-5-one	16.95	1.05	[10]-Isoshogaol
2-Butanone, 4-(4-hydroxy-3-methoxyphenyl)-	10.88	1.00	Zingerone
3-Decanone, 1-(4-hydroxy-3-methoxyphenyl)-	14.75	0.96	Paradol
5-Hydroxy-1-(4-hydroxy-3-methoxyphenyl)decan-3-one	15.66	0.85	(S)-(+)-[6]-Gingerol
1-(4-Hydroxy-3-methoxyphenyl)decane-3,5-dione	15.35	0.52	(8)-Gingerdione
(E)-1-(4-Hydroxy-3-methoxyphenyl)dodec-3-en-5-one	15.86	0.39	[8]-Isoshogaol
(E)-1-(3,4-Dimethoxyphenyl)dec-4-en-3-one	15.22	0.33	[6]-Ishogaol
(E)-1-(4-Hydrexadicethoxyphenyl)hexadec-4-ds.3-one	18.70	0.22	(E)-[12]-shogaol
1-(4-Hydroxy-3-methoxyphenyl)dodecan-3-one	15.93	0.15	[8]-Paradol
Benzaldehyde, 4-hydroxy-3-methoxy-	8.91	0.14	Vanillin
1-(4-Hydroxy-3-methoxyphenyl)dodecane-3,5-dione	16.48	0.12	[8]-Gingerdione

**Table 5:** Results of microbiological tests of composites: reference sample (Ref.), with the addition of horseradish (GFRC_CH), ginger (GFRC_I), and cistus (GFRC_CZ). D = (log number of microorganisms*_t_*_= 0_ − log number of microorganisms*_t_*_= 24 h_).

Sample	Log (CFU/cm^2^)
	D
***E. coli***	
Ref.	0.07
GFRC-CH	0.31
GFRC-I	1.49
GFRC-CZ	1.51
***S. aureus***	
Ref.	0.24
GFRC-CH	−0.61
GFRC-I	−0.27
GFRC-CZ	2.13
***C. albicans***	
Ref.	0.35
GFRC-CH	0.37
GFRC-I	0.40
GFRC-CZ	0.81

**Table 6:** Flammability requirements for railway seat elements in accordance with PN-EN 45545-2.

Requirement R6 of PN-EN 45545-2			
	MARHE [kW/m^2^]	Ds-4	VOF-4 [min]
HL1	90	600	1200
HL2	90	300	600
HL3	60	150	300

## Data Availability

Data are contained within the article.
